# Molecular mechanisms of exercise contributing to tissue regeneration

**DOI:** 10.1038/s41392-022-01233-2

**Published:** 2022-11-30

**Authors:** Jibao Chen, Ren Zhou, Ye Feng, Lin Cheng

**Affiliations:** 1 Shanghai YangZhi Rehabilitation Hospital (Shanghai Sunshine Rehabilitation Center), Tongji University School of Medicine, Shanghai, China; 2 Shanghai Institute of Hematology, State Key Laboratory of Medical Genomics, National Research Center for Translational Medicine at Shanghai, Ruijin Hospital, Shanghai Jiao Tong University School of Medicine, Shanghai, China

**Keywords:** Medical research, Molecular biology

## Abstract

Physical activity has been known as an essential element to promote human health for centuries. Thus, exercise intervention is encouraged to battle against sedentary lifestyle. Recent rapid advances in molecular biotechnology have demonstrated that both endurance and resistance exercise training, two traditional types of exercise, trigger a series of physiological responses, unraveling the mechanisms of exercise regulating on the human body. Therefore, exercise has been expected as a candidate approach of alleviating a wide range of diseases, such as metabolic diseases, neurodegenerative disorders, tumors, and cardiovascular diseases. In particular, the capacity of exercise to promote tissue regeneration has attracted the attention of many researchers in recent decades. Since most adult human organs have a weak regenerative capacity, it is currently a key challenge in regenerative medicine to improve the efficiency of tissue regeneration. As research progresses, exercise-induced tissue regeneration seems to provide a novel approach for fighting against injury or senescence, establishing strong theoretical basis for more and more “exercise mimetics.” These drugs are acting as the pharmaceutical alternatives of those individuals who cannot experience the benefits of exercise. Here, we comprehensively provide a description of the benefits of exercise on tissue regeneration in diverse organs, mainly focusing on musculoskeletal system, cardiovascular system, and nervous system. We also discuss the underlying molecular mechanisms associated with the regenerative effects of exercise and emerging therapeutic exercise mimetics for regeneration, as well as the associated opportunities and challenges. We aim to describe an integrated perspective on the current advances of distinct physiological mechanisms associated with exercise-induced tissue regeneration on various organs and facilitate the development of drugs that mimics the benefits of exercise.

## Introduction

Physical activity mainly refers to any bodily movement produced by skeletal muscles and results in energy expenditure, broadly encompassing exercise and sports, which have been done as part of daily living, occupation, leisure, and active transportation.^1,2^ Over the last few decades, physical activity has been convinced by clinical and experimental studies as an essential element of daily life and crucial to promote health and longevity.^3–5^ Importantly, it is reported that physical inactivity has become the fourth leading cause of death worldwide nowadays.^6^ According to 2020 World Health Organization (WHO) guidelines, it is stated that 150–300 min of moderate intensity, or 75–150 min of vigorous-intensity physical activity, or some equivalent combination of moderate intensity and vigorous-intensity aerobic physical activity should be undertaken per week.^7^ However, about one-third of adults worldwide do not meet the minimal intensity or time of physical activity recommended by WHO.^8^ Substantial evidence has shown that physical inactivity mostly has a negative impact on non-communicable diseases such as coronary heart disease,^9^ diabetes mellitus,^10,11^ cancer,^12,13^ and even mental health,^14–16^ dramatically aggravating the global health burden and shortening the life expectancy of people. Therefore, promotion of physical activity is one of the important methods to improve the quality of human lifespan.

**Fig. 1:**
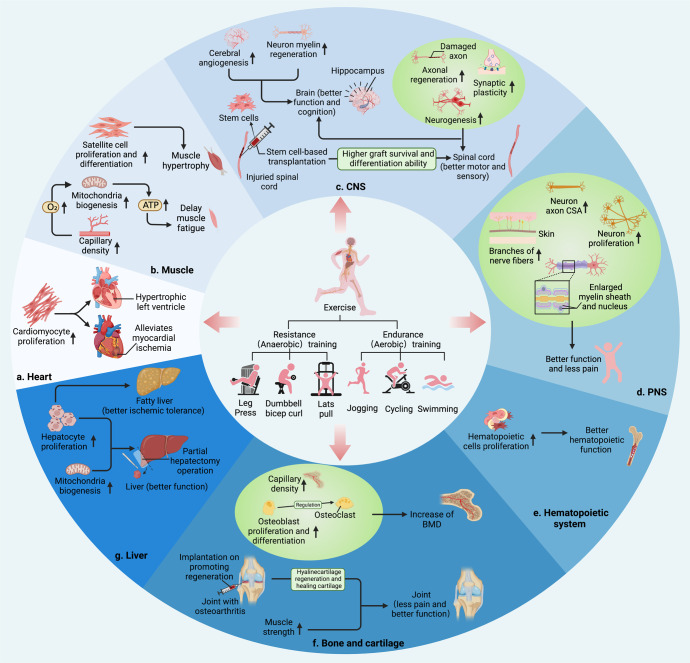
Benefits of exercise-induced tissue regeneration. **a** Exercise induces physiological hypertrophy of left ventricle and reduction of myocardial infarction area by promoting the proliferation of cardiomyocytes. **b** Exercise induces muscle hypertrophy by promoting the proliferation of the satellite cells in both physiological and pathological conditions. Angiogenesis and mitochondrial biosynthesis are helpful in delaying muscle fatigue. **c** Exercise enhances hippocampal neurogenesis, myelin regeneration, axon regeneration, and cerebral angiogenesis to improve the motor, sensory, and cognitive functions. Exercise can also improve the sensory and motor functions after spinal cord injury and promote survival and differentiation of transplanted stem cells. **d** Exercise increases the branches of nerve fibers in the proximal skin, and promotes axon cross-sectional area (CSA), myelin sheath thickness, Schwann cell’s nucleus area and neurogenesis, so as to relieve the pain, improving sensation and motor function of patients with peripheral neuropathy. **e** Exercise can change the bone marrow microenvironment, promote the proliferation of hematopoietic stem and progenitor cells and the production of leukocytes. **f** Exercise regulates skeletal stem cell to differentiate into osteoblasts and chondrocytes, as well as bone angiogenesis, increasing bone mineral density (BMD). Besides, exercise induces the regeneration of post-traumatic cartilage lesions. Exercise is also considered as an effective adjuvant to stem cell-based therapy and the application of biomaterials or devices for cartilage regeneration. **g** Exercise promotes the regeneration ability of fatty liver, improving its tolerance to ischemia. Besides, exercise can restore liver function by promoting hepatocyte proliferation and mitochondrial biosynthesis in patients with partial hepatectomy. Created with BioRender

Exercise is theoretically defined as a kind of planned, structured, and repetitive physical activity.^1^ As a kind of physical education activity and social cultural activity, regular exercise is accepted by more and more people. Basically, there are two types of exercise including endurance (aerobic) training and resistance (anaerobic) training (Fig. 1).^17–20^ Physiologically, endurance exercise training refers to the exercise in which glucose metabolism depends on oxygen under aerobic conditions, while resistance exercise training refers to the exercise in which weight or overload is carried out in anaerobic condition and it is a short period of high-intensity or maximal intensity activity.^21–23^ The former usually induces the adaptation of cardiovascular and respiratory systems, while the latter is more likely to result in muscle hypertrophy through increasing myofibrillar volume predominantly in type II fibers and it is accompanied by changes in central nervous system.^24–27^ According to the exercise testing and prescription guidelines of the American Academy of Sports Medicine,^28^ endurance exercise is a wide range of physical activities, such as walking, jogging, dancing, swimming and cycling, its intensity is lower than the maximum intensity and can last for several minutes to several hours.^29^ Endurance exercise training is based on the FITT (frequency, intensity, time, type)–VP (volume, progression) principle of exercise prescription, while the intensity of exercise can be described in terms of heart rate, oxygen consumption (VO_2_) and metabolic equivalent.^2^ Common resistance exercise training includes barbell bench press, barbell overhead squat, dumbbell bicep curl and other strength exercises. The optimal training load for strength training is still being explored.^30^ It is suggested that performing a single set of 6–12 repetitions with loads ranging from approximately 70–85% one repetition maximum 2–3 times per week may produce sufficient training effect.^31^ Indeed, the complexity of physical activities makes no such clear boundary between the two types of exercise, as endurance exercise may become anaerobic if the intensity of aerobic exercise exceeds the anaerobic threshold. With a better understanding of the physiological responses triggered by different types of exercise, a variety of exercise strategies have emerged, including high-intensity interval training (HIIT) and moderate-intensity continuous exercise training (MICT). Notably, HIIT is getting more popular among the fitness enthusiasts and athletes because it has demonstrated superiority in cardiorespiratory fitness,^32–34^ weight loss,^35,36^ and improvement of chronic diseases.^37^ Overall, the diversity of exercise triggers different physiological adaptations, allowing for the targeted utilization of exercise training to make improvements of the various physical states.

Over the past centuries, exercise training has been regarded as an effective approach to enhance tissue function under normal physiological condition and restore function under diverse pathological conditions, including cardiovascular diseases,^38–40^ chronic metabolic syndromes,^41,42^ neurological and psychiatric disorders,^43–45^ and cancer,^19,46–48^ which are mainly treated by chemical drugs. However, many diseases with loss of functional cells rely on cell supplement for tissue or organ regeneration. Nowadays, the definition of regeneration has been getting more precise and clearer, known as embryonic regulation, homeostatic regeneration, and restorative regeneration, respectively.^49,50^ As restorative regeneration stands for the ultimate goal that implantation of regenerated tissue constructs into the body to repair injuries or replace the physiological function, it makes great sense to find out how homeostatic tissues initiate the regeneration program by triggering a coherent immune response, appropriate cell plasticity, as well as stem and stromal cell responses following injury.^51^ As is well known, the basic process of tissue or organ regeneration should involve various forms of cellular plasticity first, which means that regeneration of tissues damaged by various injury modify tissue architecture through coordinated cell proliferation, differentiation, and apoptosis.^52^ Regeneration physiologically contributes to homeostasis against cell apoptosis, but it shows so limited ability to repair ischemic or impaired tissue for fully functional recovery in several vital organs, for instance cardiovascular and nervous system.^53–56^ Therefore, many approaches are currently applied to tissue regeneration therapy, including activation of endogenous stem cells and supplement with exogenous stem cells.^57–60^ Importantly, it has been suggested in recent decades that exercise is also able to contribute to promoting the restoration of damaged tissues, which provides a novel theoretical basis for tissue regeneration.

Since research on restorative regeneration has attracted more and more attention, exercise-induced tissue regeneration provides new therapeutic strategies. Recently it has been reported that only high-intensity training can stimulate changes in markers of mitochondrial respiratory function in skeletal muscle and enhance oxidative phosphorylation levels,^61^ which proves that there is a dose-dependent response to exercise duration and/or intensity again.^3^ However, not all individuals are able to achieve the level of exercise needed to experience the wide range of health benefits that exercise provides. Thus, it has been one of the most fascinating topics how exercise affects various organs and promotes injury repair, which drives researchers to mimic these effects with pharmaceuticals. To develop effective therapeutic approaches, it is crucial to understand the underlying mechanisms of the regenerative effects triggered by exercise, at the level of molecules, cells, and systems. This review will summarize recent advances in the benefits of exercise on different vital organs, thereby revealing the underlying molecular mechanisms and the potential mimetics from the perspective of exercise-induced tissue regeneration for further research and clinical applications.

## Benefits of exercise on tissue regeneration

### Exercise-induced muscle regeneration

The musculoskeletal system is an integral component of mobility and strength in the human body. Skeletal muscles, as the most important component of the musculoskeletal system, are characterized by myofibers and connective tissue, making up more than 35% of the adult body and containing 50–75% of all body proteins.^62^ The mechanical injury, ischemia injury, inflammatory injury and even congenital or acquired atrophy are described as those that involve disrupted connective tissues, leading to loss of skeletal muscle mass, reduced motor unit discharge rate, and impaired function.^63^ It has been uncovered that moderate exercise training enhances muscle regeneration after injury, as skeletal muscle mass recovery after extensive injury can be improved by contractile activity. In general, it has been widely convinced that the local adaptations of endurance exercise in skeletal muscle mostly focus on increased mitochondrial biogenesis and capillary density, which aids in the body’s ability to transport and use oxygen to generate energy and therefore delay the onset of muscle fatigue during prolonged aerobic performance.^64^ However, it has been also shown that endurance exercise training promotes maintenance of muscle mass and recovery after injury.^65,66^ While in rat models of notexin-induced soleus muscle degeneration, running exercise ensured the full recovery of muscle mass and muscle cross-sectional area during muscle regeneration, and made muscle recovery much faster than sedentary group.^67,68^ In contrast, resistance exercise training is more conducive to an increase in muscle strength and power as a result of neuromuscular adaptations, increases in muscle cross-sectional area (CSA), and alterations in connective tissue stiffness.^69^ Strength training significantly improved protracted quadriceps muscle atrophy in anterior cruciate ligament reconstruction patients via increasing muscle fibers CSA and type I fibers.^70^ Of note, it has been reported that HIIT, the novel combination exercise training strategy, promotes muscle regeneration, innervation, and vascularization in regenerated areas of volumetric muscle loss injury, even enhancing the healing effect of stem cell transplantation with an amniotic membrane scaffold.^71^ As mentioned about muscle regeneration induced by various exercise training, further studies should seek to assess different loads/modes (uphill vs. flat) with the same volume to confirm whether this affects muscle regeneration following training and provide compound exercise patterns to improve the efficiency of muscle regeneration.

Myogenesis follows after early inflammation and revascularization, and later fibrosis and re-innervation, resulting restoration of muscle mass and function.^72^ The bona fide tissue-specific stem cell, considered as a key component in myogenesis, in human adult skeletal muscle is the satellite cell.^73,74^ It has been convinced that the satellite cells are activated, proliferating, and differentiating after muscle fibers get injured, whose mitotic activity can be enhanced by exercise in the forms of endurance or resistance exercise training.^75,76^ In addition, skeletal muscle regeneration by the modulation of satellite cells is affected by the balance between pro- and anti-inflammatory macrophages.^77^ Exercise has been confirmed to cause the transition from the M1 to M2 macrophage phenotype, regulating the satellite cells proliferation and differentiation in the injury sites.^78–80^ Moreover, fibro-adipogenic progenitors (FAPs) has also been confirmed to get activated in response to muscle injury and establish functional interactions with the inflammatory cells and the satellite cells to promote muscle repair.^81–83^ More recently, it has been demonstrated that exercise increases and activates satellite cells by promoting FAPs senescence in the mouse models of acute muscle injury and chronic inflammatory myopathy, which provides a new therapeutic strategy for exercise-induced muscle regeneration (Fig. 1b).^84^

### Exercise-induced bone and cartilage regeneration

Briefly, the primary features of an ageing skeleton are loss of bone, degraded articular cartilage, and degenerate, narrowed intervertebral discs, contributing to pain and loss of mobility. Physical activity has long been recognized as an essential factor in the maintenance of skeletal health. An abundance of studies has shown that both endurance and resistance exercise physiologically promote bone growth of teenagers and increase peak bone mass, which contributes to prevention of osteoporosis in adult stages.^85,86^ Thus, it is convinced that the common decline in bone mass during ageing attenuates, by following specific exercise programs, especially in postmenopausal women.^87^ Although endurance exercise is important in maintaining overall health, the resistance training may be more applicable to the basic rules of bone adaptation and site-specific effects of exercise.^88^ Recently an 8-week of exercise protocol of resistance exercise or endurance exercise experiment confirmed resistance exercise, but not endurance exercise, is likely to be effective in increasing bone strength.^89^ Indeed, it has been confirmed that bone responds more positively to mechanical loads that induce high-magnitude strains at high rates or frequencies, such as quick jumping, which causes the weakness of exercise’s ability to evoke osteogenesis in traditional training patterns.^90^ Thus, Davison et al.^91^ established a novel exercise equipment and exercise training patterns to improve the efficiency of osteogenesis, giving hope to those afflicted with bone loss (osteoporosis, or osteopenia) conditions. In addition, bone regeneration also relies on vascularization of the ossifying tissue, called angiogenesis-osteogenesis coupling.^92^ Yao et al.^93^ found that treadmill running could physiologically increase vessel number in the proximal metaphysis of rats, and significant changes of bone mineral density (BMD) in response to exercise. Subsequently, a set of researches have clarified that exercise stimulates angiogenesis during bone defect healing, accelerating bone regeneration as well.^94,95^

Chondral injury is a pathology with high prevalence, reaching as much as 63% of general population and 36% among athletes.^96,97^ Despite inappropriate or excessive exercise primarily aggravates joint damage, moderate exercise is recognized to exert a beneficial effect on the healing of osteoarthritis. It has been widely reported that both traditional training, such as running and swimming, and non-traditional training, such as pilates and yoga, are effective in the management of knee and hip osteoarthritis, mainly regarding pain and strength improvement.^98–100^ Notably, rodent models have shown that moderate exercise prevents the progression of post-traumatic cartilage lesions.^101,102^ Additionally, it is suggested that there is a dose–response relationship between loading and intervertebral disc regenerative processes, implying that the loading pattern typically used in the lumbar extension resistance exercise interventions (high load, low volume, and low frequency) may impart the regeneration of the intervertebral discs.^103^ Actually, cartilage tissue presents limited cellularity and lacks a vascular system, leading to restrained healing capability, which brings more attention to the implantation on promoting regeneration, including stem cell transplantation and the application of biomaterials or devices.^104–106^ Importantly, exercise is also considered as an effective adjuvant to cartilage regeneration therapy. Substantial evidence has shown that exercise enhances the potential of autologous chondrocyte implants, matrix-induced autologous chondrocyte implants, and mesenchymal stem cell (MSC) implants for the successful treatment of damaged articular cartilage and subchondral bone by downregulating osteoclastogenic cytokine production and upregulating antiosteoclastogenic cytokine production by circulating immune cells.^107^ More recently, Liu et al.^108^ demonstrated exercise promoted hyalinecartilage regeneration and completely healed cartilage in osteochondral defect rabbits with a biodegradable piezoelectric scaffold implanted, which is potentially applicable to the treatment of osteoarthritis.

As is well known, physical activities induce mechanical stress to the joints and bones, promoting stem cell proliferation and differentiation in the process of regeneration.^103,109–111^ It has been reported that acute exercise increases circulating stem and progenitor cells, including hematopoietic stem cells (HSCs) and MSCs.^112^ Interestingly, osteoblasts and chondrocytes are derived from multipotent skeletal stem cells of MSCs,^113,114^ while osteoclasts are derived from the macrophage lineage of HSCs.^115^ Bone formation is carried out by osteoblasts and resorption is carried out by osteoclasts, which means that bone regeneration relies on the balance of two types of cells. Exercise has been shown to induce skeletal stem cells to differentiate towards osteoblasts. It is found that endurance training increases the total number of bone marrow MSCs in mice, enhances the osteogenic differentiation potential of MSCs, and inhibits the adipogenic potential of MSCs.^116^ Of note, osteoclast recruitment to the future resorption sites is mainly controlled by osteoblasts. It has been shown that moderate exercise increases the expression of osteoprotegerin and decreases the expression of receptor activator of nuclear factor κB ligand, both of which are expressed by osteoblasts, inhibiting osteoclast differentiation and activity.^117–119^ Thus, the regulatory mechanisms of stem cells responding to mechanical stimuli and biochemical signaling is critical for exercise-induced bone regeneration (Fig. 1f).

### Exercise-induced cardiac regeneration

The benefits of exercise on cardiovascular system have been extensively reported.^38,120,121^ It is widely accepted that both endurance and resistance exercise training contribute to larger left ventricle structures than sedentary controls from the imaging findings, presenting physiological cardiac hypertrophy.^122^ Thus, one of the most significant exercise-induced adaptations has been described as promoting cardiac growth. However, it has been discovered that the renewal of cardiomyocytes gradually decreases from 1% turning over annually at the age of 20 to 0.3% at the age of 75, implying that adult human cardiomyocytes has a limited self-renewing capacity.^53^ Importantly, Boström et al.^123^ demonstrated that adult cardiomyocytes physiologically increased in both size and proliferation rate in response to exercise in mouse models. Meanwhile, it has been identified that endurance exercise increases birth of new cardiomyocytes in adult mice (~4.6-fold) based on incorporation of 15N-thymidine by multi-isotope imaging mass spectrometry.^124^ Therefore, exercise training provides a new intervention for enhancing the proliferation of cardiomyocytes.

Interestingly, Bei et al.^125^ stated that cardiomyocyte proliferation was not necessary for exercise-induced cardiac growth but required for its protection against ischemic injury. Ischemic injury, a mismatch of oxygen and substrate supply and demand in the myocardium, is one of the most predominant causes of cardiomyocyte loss. Exercise has been found to reduce adverse ventricular remodeling and cardiac dysfunction when initiated after infarction in animal models^126,127^ and humans^128^ for decades. Furthermore, it was found that myocardial infarct size significantly decreased in ischemia injury rats at least 1 week following the cessation of 5 consecutive days HIIT training, implying the sustained capability of exercise in cardiac repair.^129^ Subsequently, a range of studies have found that exercise increases numerous circulating factors to promote cardiomyocyte proliferation and reverse pathological cardiac remodeling in post-infarction models.^130–134^ Importantly, Vujic et al.^124^ demonstrated that exercise induced a robust cardiomyogenic response in an extended border zone of the infarcted area, validating the endogenous cardiomyocyte generation induced by exercise in the process of myocardial injury repair. However, previous studies have been done in the acute phase of cardiomyocyte loss, the effect of exercise on the pathological state of the chronic phase deserves further investigation.

Although exercise has protected against pathological cardiac remodeling, the effect of myocardial restoration after ischemic injury appears to vary from different exercise types.^135,136^ Interestingly, it has been found that HIIT, the popular exercise strategy, is not superior to MICT in changing left ventricular remodeling or aerobic capacity in the heart failure patients with preserved ejection fraction^137^ or reduced ejection fraction.^138^ Additionally, recently it has been reported that moderate heart rate reduction induces cardiomyocytes proliferation under physiological conditions and promotes cardiac regenerative repair after myocardial injury by inducing G1/S transition and increasing the expression of glycolytic enzymes in cardiomyocytes, which is exactly the opposite of the exercise-induced rapid heart rate.^139^ Thus, the mechanism of exercise-induced myocardial regeneration is quite complex, the effects of different training patterns and training intensities in cardiac regeneration needs further exploration. It is rational to investigate exercise mimetics to balance the benefits and drawbacks of exercise. Furthermore, there are other causes of myocardial injury, such as hypertension^140^ and cancer,^141^ leading to cardiomyocyte death, whether exercise training counteracts induced-cell death by promoting cardiomyocyte proliferation also remains unknown (Fig. 1a).

### Exercise-induced regeneration in central nervous system

Neural stem and progenitor cells (NSPCs) are major promoters of central nervous system (CNS) regeneration, which migrate and differentiate into highly specified networks of neurons via neurogenesis, while oligodendrocytes and astrocytes are generated via gliogenesis.^142^ NSPCs response to CNS injury is extraordinarily complex and dependent upon the extent and location of injury, thus the endogenous adult neurogenesis has been highly controversial. Recently, it has been confirmed that the hippocampus contains NSPCs that continue to generate new neurons, called adult hippocampal neurogenesis (AHN), which almost continues across the lifespan, though declining with aging.^143,144^ Age-related neurodegenerative diseases are probably associated with impaired AHN. These animal studies have shown that voluntary exercise promotes hippocampal neurogenesis and prevents age-related decline in cell-proliferation in this brain structure.^145,146^ Furthermore, it has also been revealed that exercise induces volumetric retention in the left hippocampus in humans, implying endurance exercise interventions are useful for preventing age-related hippocampal deterioration.^147^ Besides aging, trauma, ischemic injury, and inflammation often bring about irreversible damage and loss of function to the CNS. Ischemic injury remains an important risk of neuron loss in the CNS. It has been reported that both endurance and resistance exercise training enhance cognitive performance^148–150^ and improve functional performance, such as balance and walking speed,^151–153^ in post-stroke population, implying that exercise promotes the repair of central neurons. Moreover, in the ischemic stroke rodent model, it has been confirmed that early endurance exercise, for instance wheel-running and treadmill training, contributes to functional and neuronal recovery, mainly improving motor function via enhancing synaptic plasticity,^154^ promoting myelin regeneration^154^ and neuron survival,^155^ and facilitating cerebral angiogenesis.^156^ Multiple sclerosis (MS) is another kind of CNS disorders characterized by oligodendrocyte loss and axonal degeneration/demyelination.^157^ Recently emerging research suggests that exercise has therapeutic benefits on the outcomes in the CNS for MS patients, including oligodendrogenesis, remyelination, and axonal regeneration.^158^

Spinal cord injury (SCI) disrupts both axonal pathways and segmental spinal cord circuitry, resulting in permanent loss of motor, sensory, and autonomic function. Exercise has been shown to induce synaptic plasticity and restore motor/sensory function in SCI patients.^159^ Subsequently, endurance exercise, such as treadmill training, has been reported to enhance axonal regeneration and sprouting in SCI rodent models via multiple ways.^160–162^ Of note, it has been revealed that the cholinergic neurotransmission from spinal locomotor neurons activates spinal NSPCs, leading to neurogenesis in the adult zebrafish. Interference with γ-aminobutyric acid signaling promotes functional recovery after spinal cord injury, which acts in a non-synaptic fashion to maintain NSPCs quiescence.^163^ Though it provides a new approach for locomotor networks’ activity-dependent neurogenesis during SCI regeneration, whether similar effects can be found in mammals needs to be further investigated. Currently, the multi-faceted regeneration strategies for SCI regeneration have been met with mixed success, however, adult neurogenesis in heterogeneous NSPC populations is still creating barriers to the function recovery of SCI. Indeed, NSPCs proliferate and differentiate into reactive astrocytes in the injured spinal cord, contributing to the glial scar border, which segregates the injury and prevents additional damage.^164,165^ However, this scar also prevents axonal outgrowth into the site of injury and generation of new cell types within the neural lesion.^166^ Thus, stem cell-based transplantation, including olfactory ensheathing cells (OECs), MSCs, and NSPCs, has opened an avenue for functional recovery of SCI, which has been enhanced by exercise training as well.^167^ It has been reported that exercise enhances the effect of OEC grafts in super acute thoracic cord transected rats, inducing a fourfold increase in regenerating axons within the caudal stump of the transected spinal cord.^168^ Additionally, exercise significantly promoted NSPCs graft survival and differentiation more into neurons and oligodendrocytes, enhancing myelination, and restoration of serotonergic fiber innervation in the lumbar spinal cord via reducing stress caused by active oxygen or active nitrogen through insulin-like growth factor 1 (IGF-1) signaling, which provided more theoretical basis for exercise rehabilitation and pharmacological mimetics^169^ (Fig. 1c).

### Exercise-induced regeneration in peripheral nervous system

The regenerative capacity of the nervous system varies considerably between the peripheral nervous system (PNS) and CNS. On the contrary, the adult human PNS retains the ability of axons to regenerate after injury and successfully reinnervate the intended target.^170^ In the PNS, injured nerves undergo successful Wallerian degeneration and subsequently the axons upstream of the injury undergo polarized growth toward their target tissues.^171^ Therefore, enhancing the regeneration of axons is often considered to be a therapeutic target for improving functional recovery after peripheral nerve injury. Several clinical studies have suggested exercise as a non-pharmacological approach to positively affect various aspects of peripheral neuropathy such as diabetic peripheral neuropathy (DPN),^172,173^ chemotherapy-induced peripheral neuropathy (CIPN),^174,175^ and even carpal tunnel syndrome.^176^ Among these studies, a 10-week composite training program of endurance and resistance exercise led to significant reductions in pain and neuropathic symptoms, and increased intraepidermal nerve fiber branching from a proximal skin biopsy in DPN patients.^173^ Similarly, the positive effects of exercise, including decreasing pain and improving physical function^177^ as well as improvement of deep sensitivity^178^ and static balance performance,^179^ have been confirmed in CIPN patients. The effect of exercise promoting peripheral nerve regeneration is also observed in animal models. Physiologically, ladder-based resistance training effectively induced similar growth in the radial and sciatic nerves (SN) of adult rats including myelinated axons CSA, unmyelinated axons CSA, myelin sheath thickness, and Schwann cells nuclei area.^180^ Meanwhile, the functional and histological recovery after the mouse SN crush was positively influenced by treatment with eccentric exercise.^181^ Moderate swimming training was found to promote nerve regeneration in SN ligation or SN transection mice as well.^182,183^ Additionally, treadmill training accentuated nerve regeneration, accelerated functional recovery and prevented muscle atrophy in median nerve crush injury rat models^184^ (Fig. 1d).

### Exercise-induced regeneration of other tissues

The liver exhibits the unique regenerative capacity that ensures body homeostasis or post-injury repair.^185^ Experimental models that involve partial hepatectomy or chemical injury have revealed the efforts of exercise that make the liver return to equivalent size and weight to those prior to injury. It has been reported that a 4 weeks endurance exercise program markedly enhances the ischemic tolerance and the regenerative capacity of fatty liver in diet-induced steatosis mice.^186^ Moreover, Fard-Aghaie et al.^187^ confirmed that a novel physical prehabilitation of treadmill training promoted hepatocyte proliferation and enhanced mitochondrial biogenesis, restoring liver function after partial hepatectomy operation in rodent models. Although the liver is a solid organ with a high regenerative capacity, the rate of physiological cell turnover is very slow. Thus, the regenerative activities of hepatocytes and cholangiocytes induced by exercise provides new strategies for restoring liver function (Fig. 1g).

The skin, the largest organ of the human body, defends against daily assaults from the external environment. In general, scarring and regeneration are two physiologically opposite endpoints to skin injuries. Thus, scarless regeneration is the ultimate goal in repairing skin injury. Indeed, emerging evidence has shown exercise promotes skin wounds healing. For example, endurance exercise improves cutaneous wound healing rates of different etiologies in mice and humans.^188–190^ Interestingly, a study of mice trained on a motorized treadmill has suggested that different intensities of exercise have different impacts on healing rates.^191^ In addition, another study has shown that low-intensity exercise accelerates wound healing rates in diabetic mice but high-intensity exercise fails.^192^ However, promoting wound healing is not the same as promoting skin regeneration, as there are few studies for further cellular mechanisms. Whether exercise promotes resident skin stem cell proliferation remains unclear.

Exercise offers regenerative effects to the hematopoietic system as well. In this regard, preclinical studies of treadmill exercise training in mouse models have demonstrated endurance exercise is able to modify the bone marrow microenvironment, alter hematopoiesis, and accelerate hematopoietic regeneration.^193,194^ Leukocytes derive from hematopoietic stem and progenitor cells (HSPCs), acting as a major responder of exercise. Recently it has been reported that running exercise diminishes leptin production in adipose tissue to regulate HSPCs proliferation and leukocyte production in mice.^195^ The impact of exercise on leukocyte production and on HSPC epigenome and transcriptome persists for several weeks. Curiously, it was also showed that ultra-endurance exercise contributed to an increase in circulating leukocytes and induced an inflammatory response that resulted in a highly significant decline of circulating hematopoietic progenitor cells functionality in humans.^196^ This may be the result of different inflammatory effects of the organism in response to different exercise intensities and patterns. Hematopoietic stem cell transplantation (HSCT) is increasingly used for hematological malignancies or severe non-malignant hematological disorders. Since exercise is convinced to affect HSPC, HSCT combined with exercise therapy is also a topic worth being explored to improve prognosis.^197^ Lisio et al.^198^ demonstrated a survival benefit and increased total blood reconstitution in mice that were pre-conditioned with endurance exercise after bone marrow transplantation. However, as implementation varies across studies in terms of timing of exercise initiation, exercise types and duration of exercise according to potential benefits of exercise reported by previous studies, the efficacy of exercise program among HSCT patients varies.^199,200^ The efficacy of exercise promoting HSCT is worthy of recognition, however, further mechanistic studies are needed (Fig. 1e).

## Molecular mechanisms of tissue regeneration induced by exercise

In response to exercise, the organism will have both structural and functional adaptive changes which confer the beneficial effect of exercise. However, the mechanisms by which exercise initiates cellular responses involved in tissue repair/regeneration are still inadequately understood. Generally, exercise predominantly leads to an increase in mechanical signals such as fluid flow, dynamic tension, compression, and hydrostatic pressure. Thus, mechanotransduction, the process by which the organism converts mechanical loading into cellular responses, is regarded as one kind of potent signaling pathways for adaptive responses to exercise.^201^ Besides mechanical signals, numerous studies have shown that a range of bioactive substances regulated by exercise (namely, exerkines) contribute to maintaining homeostasis and improving the impaired function of diverse organs.^202,203^ These exerkines can be secreted by a variety of cells, including satellite cells, osteoblasts, immune cells, endothelial cells, fibroblasts, macrophages, and even adipocytes, which act as autocrine, paracrine, or circulating regulators in response to exercise.^204,205^ Notably, it has been extensively investigated that both mechanical stress and biochemical signals are involved in promoting cell proliferation and renewal induced by exercise.^206–210^ Since the complicated mechanism of exercise-induced tissue regeneration advances rapidly, we will discuss in detail the substantial novel progress in the regulation of tissue regeneration from multiple aspects.

### Mechanotransduction

Indeed, a variety of cell surface proteins and structures, named mechanosensors, has been proposed to convert these mechanical stimuli into electrical or biochemical signals. The Piezo family, one of the mechanically activated ion channel, has emerged as the critical mechanosensors in many cell types, responding to various forms of mechanical forces, including membrane stretch, static pressure, and fluid shear stress.^211–213^ Of note, Piezo1 is also highly expressed in osteocytes and can be upregulated by mechanical stretching, involved in stem cell differentiation and bone formation.^214–216^ In addition, Piezo1 channels, working as non-selective cationic channels in endothelial cells, had profound importance for shear stress-evoked Ca^2+^ signaling, sensing the exercise induced changes in blood flow.^217^ Focal adhesions, one type of integrin-based adhesion complex, is another important mechanosensor of transmitting mechanical signals and promoting protein biosynthesis.^218–220^ Unlike integrin-based adhesions that receive mechanical stimuli from the extracellular matrix, gap junctions, two juxtaposed connexons on the surfaces of adjacent cells, are bridges for mechanical signaling communication between cells. Multiple types of connexins play a role in responding to the mechanotransduction, among which connexin 43 was shown that its knockout in early osteoblasts caused impaired muscle formation in mice.^221,222^ Moreover, low density lipoprotein receptor-related proteins 5/6 (LRP5/6), the single-pass transmembrane protein, have been found to act as the receptor of Wnt ligands and be indispensable for Wnt/β-catenin signaling transduction, which has been shown to affect bone mass by regulating osteoblast proliferation and activity.^223–225^ Besides these canonical membrane structures, novel mechanosensitive proteins are regularly discovered, such as the recent discovery of plexin D1 as a mechanosensitive receptor,^226^ with more likely awaiting discovery. Taken together, these important membrane structures are the cornerstone of various cellular responses to external mechanical stimuli, and may be another prelude to unlocking the secrets of exercise induced regeneration.

**Fig. 2:**
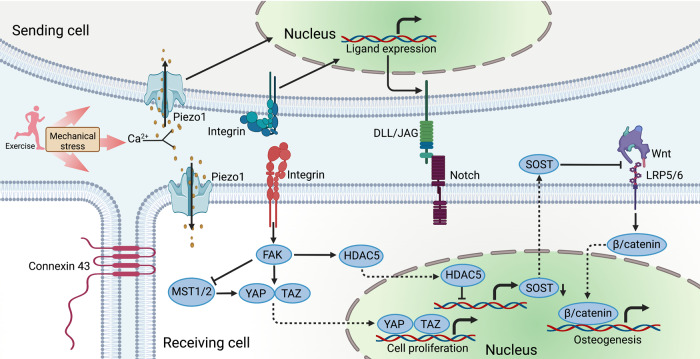
Mechanotransduction regulating regenerative responses. The signaling transduction of exercise-induced regeneration are generally initiated by mechanical signals. A series of membrane receptors or channels can respond to mechanical signals, thereby converting mechanical signals into chemical molecular signals. Piezo1 channels, working as non-selective cationic channels, sense the exercise-induced stress, converting into mechanical stress-evoked Ca^2+^ signaling. Integrin-based adhesion complex activates FAK, promoting YAP/TAZ translocation via inhibiting Hippo signaling. FAK can drive HDAC5 nuclear translocation and suppress SOST. Integrins or Piezo channels activate the transcription of ligands of Notch receptor in sending cell, triggering Notch signaling of receiving cell. Wnt/β-catenin pathway is important in osteogenesis, which can be inhibited by SOST. Connexin 43 responds to the mechanotransduction for cell-to-cell communication. Created with BioRender

Within the help of the mechanosensors, the activation of sequential signaling cascades and expression of downstream target genes exhibit some common features, even in various cell types, including osteocytes,^227^ myocytes,^228^ neurons,^229,230^ liver cells,^231,232^ and cardiomyocytes.^233,234^ Basically, signal transduction can occur through the direct physical connections between the membrane, the cytoskeleton, and the nucleus, triggering gene expression and protein synthesis.^235^ Importantly, the transmission of information still involves facilitating biochemical signals via intracellular signaling molecules and secondary messengers. The activation of Wnt/β-catenin pathway has been proven to be a key regulator of cell growth. Recently a multi-omic analysis of stretched osteocytes has uncovered mechanically stimulated osteocytes support bone regeneration via ossification and extracellular matrix remodeling, focusing on the activation of Wnt/β-catenin pathway in both human and murine cells, showing the conservation of mechanotransduction mechanisms.^236^ It has been established that exercise-induced loading reduces expression of sclerostin (SOST) and Dickkopf-related protein 1 (Dkk1), the inhibitors of the Wnt pathway, in osteocytes, thus stimulating new bone formation.^237^ Focal adhesion kinase (FAK), an attachment protein associated integrin-based adhesion complex, has also been a key component of transmitters of mechanical signals. FAK was reported to be required for IGF-1-induced muscle hypertrophy, through tuberous sclerosis complex 2 (TSC2)/mammalian target of rapamycin (mTOR)/ribosomal S6 kinase 1 (S6K1)-dependent signaling pathway.^238^ Interestingly, fluid flow shear stress could trigger FAK dephosphorylation, driving class IIa histone deacetylase 5 (HDAC5) nuclear translocation, which demonstrated a role for HDAC5 in loading-induced SOST suppression.^239^ Another most described mediators are the transcriptional coactivators yes-associated protein (YAP) and transcriptional coactivator with PDZ-binding motif (TAZ).^240^ Generally, YAP and TAZ, known to be regulated by FAK through RhoA-mediated contractile force, translocate from the cytoplasm to the nucleus in cells perceiving high levels of mechanical signaling.^241,242^ Notably, nuclear translocation of YAP/TAZ has been also regulated by Hippo signaling pathway.^243,244^ Multiple mechanical stimuli mostly inhibit the Hippo pathway, promoting YAP/TAZ to enter the nucleus to activate genes involved in the cell cycle and cell proliferation.^245,246^ Thus, suppression of Hippo signaling play an important role in promotion of tissue regeneration as well, suggesting a new intervention mechanism for exercise-induced regeneration.^247,248^ Furthermore, Notch signaling has been shown to be another associated pathway of mechanotransduction as well.^249^ Generally, mechanical loading sensed by integrins or mechanosensitive Piezo results in the transcription of ligands of sending cell, regulating Notch signaling of receiving cell, which modulates cell proliferation and differentiation.^250^ It has been reported that Notch signaling is impaired in regenerating aged skeletal muscle, which can be restored by physiological stimuli of exercise.^251–253^ Therefore, further researches should be carried out to demonstrate the underlying mechanisms of mechanotransduction mediated tissue regeneration with different exercise types, which may provide the novel targets for clinical interventions (Fig. 2).

### PI3K/Akt signaling pathway

Emerging evidence has revealed the essential contribution of PI3K/Akt signaling pathway to exercise-induced regeneration.^254,255^ An array of growth factors has been reported to act as exerkines, such as IGF-1,^256^ brain-derived neurotrophic factor (BDNF),^257^ epidermal growth factor (EGF)^258^ and their associated family, triggering the cellular responses via PI3K/Akt pathway. Amongst all the growth factors induced by exercise, the most widely studied is IGF-1. Notably, circulating IGF-1 is primarily secreted by the liver, while peripheral tissues, including bone and muscle, produce IGF-1 as well, acting in a paracrine/autocrine fashion. Exercise-induced IGF-1 locally leads to the sequential activation of PI3K/Akt signaling pathways with consequent induction of myoblasts,^259,260^ osteocytes^261,262^ and cardiomyocytes^263,264^ proliferation and differentiation. Meanwhile, neuregulin 1 (NRG1), a member of EGF family, and its tyrosine kinase receptor ErbB family is found to promote cell growth and differentiation physiologically and pathologically by targeting PI3K/Akt pathway.^265–267^ Moreover, BDNF and its receptor, tropomyosin-related kinase B (TrkB) mediate PI3K/Akt pathway as well, sharing the similar effects.^268,269^ BDNF has been discovered to be time-dependently upregulated in rat skeletal muscle after acute endurance exercise, which is convinced to be involved in exercise-induced skeletal muscle regeneration.^257^ Briefly, exercise-induced BDNF expression also plays a crucial role in neuronal survival, proliferation, maturation, and outgrowth in both the brain,^270–273^ spinal cord,^274,275^ and PNS.^276,277^ Angiogenesis has also played a vital role in human physiology of tissue repair, since oxygen supply and nutrients constitute important primitive materials for tissue anabolic activity.^278–280^ The transcription of vascular endothelial growth factor (VEGF) a kind of pro-angiogenic factor, is mainly activated by exercise-induced hypoxia and mechanical stress, which plays a crucial role in endothelial cell survival and promotion of capillary sprouting via PI3K/Akt pathway.^281,282^ Several studies have revealed that both endurance and resistance exercise increase the expression of VEGF in the brain, heart, skeletal muscle, and bones.^95,283,284^ HIIT training contributed to an overall increase in the expression levels of VEGF and VEGF receptor-2 (VEGFR2) in skeletal muscle of subjects with peripheral myopathy associated with heart failure, promoting muscle capillarization,^285^ and differentially expressed genes of the skeletal muscle showed the PI3K/Akt signaling pathway was activated in response to HIIT.^286^ Of note, it has been found that the PI3K/Akt axis is not only activated by a range of exerkines mentioned above, but also in response to mechanical stress.^287–289^ Exercise frequently mediates crosstalk between mechanoregulation of regeneration and canonical regenerative signaling pathways. While Akt has been reported to be activated by Notch, a important role in mechanotransduction, in EPCs after endurance exercise in hypertension patients, targeting endothelial nitric oxide synthase, for restoration of impaired angiogenesis capacity of late EPCs.^290^

**Fig. 3:**
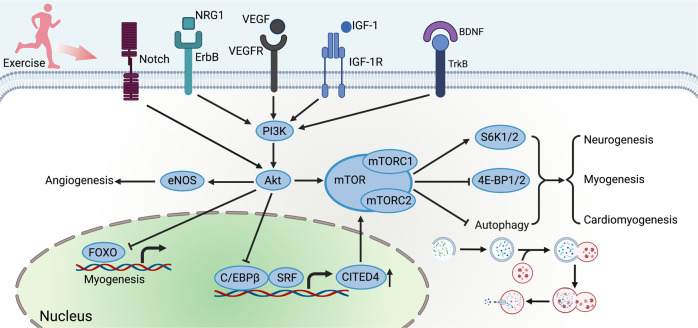
PI3K/Akt signaling pathway. The PI3K/Akt pathway is mainly activated by an array of growth factors, such as IGF-1, NRG1, VEGF, and BDNF. Akt can be directly activated by Notch signaling. The PI3K/Akt pathway mainly acts as the upstream of mTOR activation. The activation of mTOR promotes cardiomyogenesis, myogenesis, and neurogenesis via various signaling. Importantly, PI3K/Akt axis inhibits C/EBPβ, thus upregulating CITED4 to activate mTOR and promoting myogenesis. Additionally, the PI3K/Akt axis inhibits FOXOs, promoting myogenesis. eNOS is upregulated by the PI3K/Akt axis, promoting angiogenesis. Created with BioRender

The downstream responses of PI3K/Akt pathway are also varied. One of the significant targets of Akt is mTOR, an evolutionarily conserved serine/threonine kinase. Actually, mTOR exists in two distinct complexes: mTOR complex 1 and mTOR complex 2 (mTORC1 and 2).^291^ The activation of mTORC1 is frequently spotted in the adaptive response to exercise.^292^ Importantly, the activation of mTOR-axis has been the critical process of exercise-induced regeneration in different tissues, including muscle,^293,294^ heart,^295^ brain^296^ and spinal cord.^297^ Mostly, mTORC1 phosphorylates and activates S6K1/2 and eukaryotic translation initiation factor 4E (eIF4E)-binding proteins 1 and 2 (4E-BP1/2), which contribute to stimulation of mRNA translation, thereby regulating increases or decreases in anabolic and catabolic processes.^298^ Additionally, the activation of mTORC1 suppresses autophagy and perhaps other lysosomal functions.^299^ For instance, NRG1 activated PI3K/Akt axis, targeting mTOR-pathway in hippocampal neurogenesis, which was confirmed by exercise-induced expression of autophagy-related proteins.^300^ Moreover, CCAAT/enhancer binding protein β (C/EBPβ)-Cbp/p300-interacting transactivator with ED-rich carboxy-terminal domain 4 (CITED4) axis has been identified as critical modulator in the cardiomyocyte proliferation in adult exercised hearts, which has also been shown to be regulated by Akt.^123,301^ It has been confirmed that C/EBPβ is downregulated by endurance exercise to enhance cardiomyocyte proliferation via negatively regulating CITED4 in vitro.^123^ C/EBPβ and CITED4 has been reported to be regulated in myocardial ischemia or transverse aortic constriction murine models after exercise training.^39,302,303^ Overall, CITED4 acts as downstream of C/EBPβ, thereby activating the mTOR pathway, promoting exercise-induced cardiomyocyte proliferation and protecting from adverse cardiac remodeling.^303–305^ Another potential downstream mediator of the cell proliferation and development induced by exercise are forkhead box class O family (FOXOs). Akt/FOXO3a signaling pathway is activated in exercise-induced autophagy, which is beneficial for remedying sarcopenia.^306^ Akt is also likely to mediate FOXO family inhibition in the regulation of stem cell proliferation.^307^ Inhibition of FOXOs activity decreases myostatin expression and increases satellite cell proliferation, and fusion, and leads to muscle hypertrophy^308,309^ (Fig. 3).

### MAPK signaling pathways

**Fig. 4:**
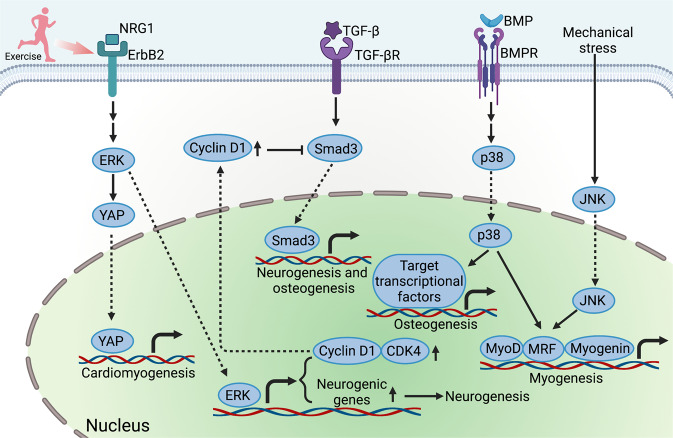
MAPK signaling pathway. The MAPKs signaling can be activated by exercise-induced mechanical stress and a range of growth factors, including NRG1, TGF-β and BMP, thus playing an important role in exercise-induced tissue regeneration. MAPK/ERK signaling activates YAP translocation into nucleus, promoting cardiomyogenesis. The activation of ERK also promotes neurogenesis and cycling of satellite cells. The restoration of cyclin D1 inhibits TGF-β/Smad signaling. The p38 MAPK and JNK can activate the transcription factors that initiate the expression of osteogenetic and myogenetic genes, including Myod, MRF, and Myogenin, promoting osteogenesis and myogenesis. Created with BioRender

The extracellular signal-regulated kinases 1 and 2 (ERK1/2), p38 mitogen-activated protein kinase (MAPK), and c-Jun N-terminal kinase (JNK), as key members of MAPK family, are also serine/threonine protein kinases that are irreplaceable players participating in diverse biological activities.^310^ Similar to PI3K/Akt signaling, MAPK signaling can be activated by a range of growth factors, thus playing an important role in exercise-induced tissue regeneration. More recently, it has been investigated that NRG1/ErbB2 signaling mediates the interaction of YAP with nuclear-envelope and cytoskeletal components for cardiomyocytes regeneration via the activation of ERK.^311^ Besides, MAPK/ERK signaling is also targeting the regulation of the cell cycle. Postnatal cell cycle exit is often accompanied by reduced expression of cyclins and cyclin-dependent kinases.^312–314^ Endurance exercise up-regulated cyclin-dependent kinase 4 and Cyclin D1 by ERK signaling, inducing the proliferation and differentiation of endogenous neural stem cells, and improving neural function of rats with cerebral infarction.^315^ Exercise was also reported to activate MAPK/ERK signaling to promote cycling of satellite cells.^294^ Furthermore, running exercise accelerated muscle regeneration in aged mice, suppressing transforming growth factor-β (TGF-β)/Smad3 signaling in quiescent muscle stem cells via the restoration of Cyclin D1.^316^ Additionally, it has also been well-known that bone morphogenetic proteins (BMPs), members of TGF-β super family, are upregulated in bones and cartilages after exercise.^317–319^ Mostly, the regulation of downstream networks of BMPs signaling is specifically though canonical Smad-dependent pathways.^320^ Importantly, BMP signaling has played a vital role in osteoblast differentiation, which promotes osteoblastogenesis through p38 MAPK pathway as well.^321–323^ Furthermore, following repeated bouts of eccentric cycling, it was reported that phosphorylation of JNK and p38 MAPK were also activated in skeletal muscle, inducing the overexpression of MyoD, myogenic regulatory factors (MRFs) and Myogenin^324^ (Fig. 4).

### AMPK/SIRT1/PGC-1α signaling pathway

**Fig. 5:**
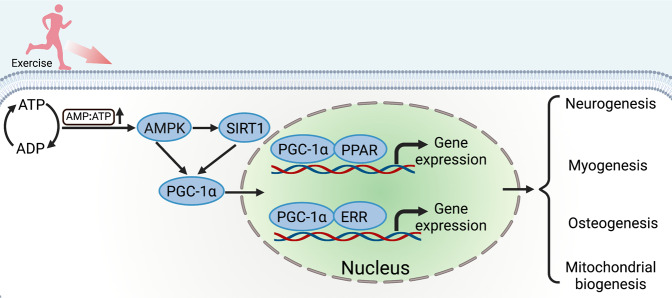
AMPK/SIRT1/PGC-1α signaling pathway. Exercise changes the energy status, consuming a large amount of ATP, which elevates AMP-to-ATP ratio. Consequently, AMPK/SIRT1/PGC-1α signaling pathway is activated. PGC-1α is very important in the anabolic process of exercise-induced response. PGC-1α and PPAR/ERR form co-transcriptional complexes that initiate the overexpression of target genes, thereby promoting tissue regeneration and repair, including neurogenesis, myogenesis, and osteogenesis. Created with BioRender

Peroxisome proliferator-activated receptor (PPAR)-γ coactivator-1α (PGC-1α) is commonly expressed in high-energy-demanding tissues such as heart, muscle, and brown adipose tissue, which is already considered as the core regulator of metabolic regulating pathways such as the adenosine monophosphate-activated protein kinase (AMPK)-sirtuin 1 (SIRT1)-PGC-1α pathway.^325^ Undoubtedly, exercise leads to the activation of AMPK in vivo through the modulation of the AMP-to-ATP ratio.^326,327^ Though PGC-1α mainly involved in mitochondrial biosynthesis and cellular respiration, has been reported to act as a vital regulator in cell proliferation and differentiation as well.^67,328^ Exercise strongly induces overexpression of PGC-1α in both human and rodent muscle,^329,330^ which may trigger a remodeling of the satellite cells niche by altering the extracellular matrix composition, including the levels of fibronectin, thus affecting the proliferative output of satellite cells.^331^ While in terms of osteogenesis, PGC-1α has already been shown to play an important role in skeletal homeostasis by coactivating a range of transcription factors.^332^ Overexpression of PGC-1α was sufficient to enhance osteocytic gene expression in IDG-SW3 cells, murine primary osteoblasts, and osteocytes, and ex vivo bone cultures.^333^ In addition, deletion of PGC-1α suppressed differentiation and activity of osteoblast, resulting in a significant decrease of cortical thickness and trabecular thickness.^334^ Recently, it has been found that running exercise increases the expression of PGC-1α in the hippocampus of depressed mice, targeting for antidepressant treatment via promoting the proliferation parvalbumin-positive interneurons.^335^ Mechanically, PGC-1α activates a variety of metabolic programs in different tissues through its ability to form heteromeric complexes with many nuclear hormone receptors, such as PPARs^336^ and estrogen-related receptors (ERRs).^337^ Interestingly, PGC-1α is regulated by AMPK/SIRT1 axis, promoting exercise-induced tissue regeneration, as well as involved in mitochondrial signaling,^338,339^ Thus PGC-1α has acted as a vital regulator in the adaptive response to exercise, which may be the key regulator of the cross-talk between mitochondrial biogenesis and exercise-induced regeneration (Fig. 5).

### Noncoding RNAs and their regulated signaling pathways

Recently emerging evidences support the critical role of noncoding RNAs, another important cluster of exerkines, in the regulation of exercise-induced tissue regeneration. Among them, microRNAs (miRNAs) have shown significant changes in the musculoskeletal system after exercise training.^340^ It was reported that in healthy untrained males, miR-1, miR-133, and miR-181a were increased in skeletal muscle samples collected 3 h following 60 min of cycling at 70% of VO_2_ peak.^341^ Similarly, the former two were shown to decrease in skeletal muscle miRNA profiles of muscle atrophy mice.^342,343^ Indeed, miR-1 promotes myoblast differentiation, whereas miR-133 stimulates myoblast proliferation. Mechanically, miR-1 modulated myocyte enhancer factor 2 (MEF2) via suppressing HDAC4 and miR-133 inhibited serum response factor (SRF), promoting myogenesis in different stages.^344^ Of note, miR-1 also positively promoted the protein synthesis and myogenesis by targeting IGF-1/Akt/FOXO3 signaling pathway.^345,346^ More recently, liver-derived extracellular vesicle miR-122-5p after treadmill training was reported to promote angiogenesis through shifting substrate preference to fatty acids in endothelial cells by targeting 1-acyl-sn-glycerol-3-phosphate acyltransferase (AGPAT1), increasing capillary density in the quadriceps, and accelerating wound healing in mice.^347^ In addition, treadmill exercise training has been also reported to influence the miRNA profiles of bone tissue, such as miR-190a-5p, miR-203-5p, miR-27a-5p, and miR-5118.^348^ Importantly, miR-27a-5p is confirmed as a member of miR-23a cluster. The components of the miR-23a cluster regulated osteoblast differentiation by targeting the modulation of SOST via TGF-β signaling pathway, which may explain the regulation of exercise-induced osteogenesis by miRNAs.^349^ Moreover, more miRNAs have been identified to involve in the different process of fracture healing, thus, whether these miRNAs are regulated by exercise still deserves further exploration.^350^

Likewise, a wide range of miRNAs have been found to increase in exercised heart as well, acting as vital regulators of exercise-induced cardiomyocyte proliferation and involving in myocardial injury repair. Expression of the protein kinase HIPK1 was identified as a direct target of miR-222 with anti-proliferative effects in cardiomyocytes, contributing to exercise-induced cardiomyogenesis.^124,131^ MiR-17-3p positively regulated cardiomyocytes proliferation and hypertrophy by targeting tissue inhibitor metallopeptidase 3 (TIMP3) and acting upstream of the phosphatase and tensin homolog deleted on chromosome 10 (PTEN)/Akt signaling pathway, protecting against myocardial ischemia–reperfusion injury.^133^ Additionally, aerobic exercise training increased miR-26 and decreased miR-16, significantly promoting cardiomyocyte hypertrophy and proliferation via Akt/mTOR signaling pathway.^351^ Furthermore, miR-34a, regulated by adenosine deaminases acting on RNA 2 (ADAR2), contributed to exercise-induced cardiomyocyte proliferation, targeting SIRT1, Cyclin D1 and Bcl2.^352^

While in terms of exercise-induced adult neurogenesis, it was reported that running exercise downregulated miR-135a-5p, targeting inositol 1,4,5-trisphosphate (IP3) signaling, thus increasing proliferation of neural precursor cells of the mouse dentate gyrus.^353^ It was also shown that miR-199a-3p increased in spinal cord after SCI and miR-21 increased in SCI animals that had undergone exercise.^354^ It was confirmed that miR-21 promoted and miR-199a-3p attenuated neurite growth in sciatic nerve injury rats via targeting PTEN in the regulation of Akt/mTOR pathway.^297^

**Fig. 6:**
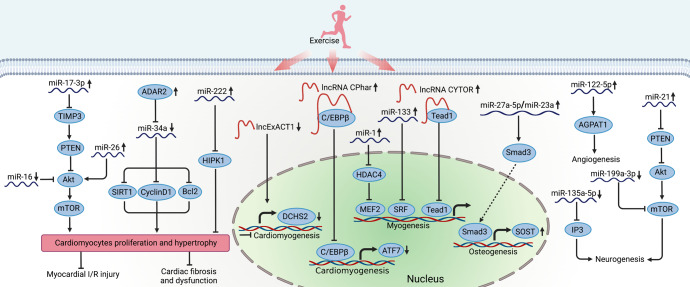
Noncoding RNA regulating the exercise-induced regeneration. Exercise induces many noncoding RNAs in the regulation of physiological response. MiR-17-3p acts as the upstream of PTEN by inhibiting TIMP3, promoting cardiomyocyte proliferation and hypertrophy through Akt/mTOR pathway. Another two miRNAs, miR-26, and miR-16, also involve in the Akt/mTOR pathway. ADAR2 inhibits miR-34a, targeting SIRT1, CyclinD1, and Bcl2 and leading to cardiomyocyte proliferation. Exercise-induced miR-222 decreases the expression of HIPK1, promoting cardiomyogenesis. Furthermore, miR-1 and miR-133 promote myogenesis via inhibiting HDAC4 and SRF, respectively. MiR-122-5p can promote angiogenesis through AGPAT1 pathway. Additionally, miR-27a-5p upregulates the expression of SOST to achieve osteogenesis. Of note, miR-21 and miR-199a-3p are also regulated by exercise, which is involved in PTEN/Akt signaling pathway. While miR-135a-5p promotes neurogenesis via inhibiting IP3 pathway. Exercise also modulates the effect of lncRNA on tissue regeneration. LncRNA CYTOR can achieve myogenesis through sequestering Tead1. LncExACT1 induces pathological myocardial hypertrophy, which is downregulated by exercise. While lncRNA CPhar promotes cardiomyogenesis through decreasing ATF7 by sequestering C/EBPβ. Created with BioRender

Similarly, long noncoding RNAs (lncRNAs) have been shown to respond to exercise as well, promoting cell proliferation and differentiation during tissue regeneration.^355^ A set of lncRNAs have been reported to play key roles in myogenesis and adult skeletal muscle regeneration.^356^ Amongst them, the overexpression of lncRNA CYTOR, responding to exercise in both human and rodents, in mouse myogenic progenitor cells enhanced myogenic differentiation by sequestering the transcription factor Tead1, which was a regulatory mechanism of fast-twitch myogenesis in aging.^357^ Meanwhile, lncRNA CPhar^358^ and lncExACT1^359^ have been found to be regulated in exercised heart. Notably, lncRNA CPhar, characterized to be increased with exercise, triggered exercise-induced cardiac physiological hypertrophy via sequestering C/EBPβ and downregulating activating transcription factor 7 (ATF7), thus preventing myocardial ischemic injury-induced cardiac remodeling and dysfunction.^358^ Whereas lncExACT1 increased in heart failure but decreased in exercised hearts, inhibition of which induced cardiomyogenesis and protected against cardiac fibrosis and dysfunction as well.^359^ Dachsous cadherin-related 2 (DCHS2) had a role in the heart as a downstream effector of lncExACT1, mainly targeting Hippo/YAP signaling.^359^ Although evidence is still lacking to support the comprehensive functionality of most lncRNAs, the high tissue-specificity and regulation of specific facets of cellular networks have suggested that lncRNAs are superior to proteins in terms of potential, undesired toxic effects associated with their targeting^360^ (Fig. 6).

## Potential exercise mimetics contributing to tissue regeneration

As is mentioned that exercise induces a set of physiological responses that benefit regeneration on various organs, it has gained great potential for use in patients having low exercise compliance or in those for whom regular exercise is not feasible. Consequently, exercise mimetics may have therapeutic applications across a variety of diseases. The following part reviews candidate exercise mimetics with emerging therapeutic targets and strategies for the development of exercise mimetics.

### Pharmaceuticals

Indeed, AMPK/SIRT1/PGC-1α pathway acts as an important role of exercise-induced physiological responses. Given it, AMPK agonists are proposed as the promising exercise mimetics.^361–363^ However, it is widely recognized that AMPK activation induces a switch of cellular metabolism from anabolic to catabolic, promoting ATP conservation by inhibiting cell growth and proliferation, which makes AMPK agonists specialize in anti-tumor therapy rather than regeneration.^364–367^ Nevertheless, 5-aminoimidazole-4-carboxamide ribonucleotide (AICAR), one of the AMPK agonists, promotes angiogenesis in muscle by activating AMPK signaling in endothelial cells, mimicking the effects of exercise.^368,369^ Interestingly, though the majority of the effects of AICAR on skeletal muscles are AMPK-dependent, it may have indirect effect of AMPK activation in other organs. For example, AICAR has been suggested to increase hippocampus neuron number via activating the overexpression of BDNF, improving spatial memory; however, it cannot maintain a sustained positive effect as well as running due to the poor permeability through the blood–brain barrier.^370–372^ In addition, AICAR acts as an exercise mimetic in settings of fatty liver disease, enhancing the ischemic tolerance and the regenerative capacity of fatty liver.^186^ Likewise, another AMPK agonist exhibiting impressive exercise mimicking capability is metformin, which is extensively used as a first-line antiglycemic drug.^373^ Metformin showed positive cognitive effects or increased memory function via promoting angiogenesis, AHN, and remyelination in aged or stroke rodent models.^374–376^ Besides neurogenesis, metformin also has exhibited capability of osteogenesis, inducing the similar effects on femoral BMD gains compared to plyometric exercise in ovariectomized rats.^377^

PPARs are proposed to interact with PGC-1α, promoting a series of exercise-induce responses. Deficiency of PPARδ has been reported to result in a reduction of satellite cell number and the regenerative capacity.^378^ PPARδ increased the proliferation and differentiation of myoblasts through FOXO1, whereas GW501516, a kind of synthetic PPARδ agonist, promoted the processes of myogenesis.^379,380^ More recently, GW501516 was also reported to limit muscle tissue damage and restores muscle tetanic contraction in mice via mimicking localized exercise-induced inflammation by upregulating Forkhead box A2.^381^ Notably, GW0742, another synthetic PPARδ agonist, promoted angiogenesis and cell proliferation in muscle^382^ and heart^383^ via activation of calcineurin. Similar to AICAR and metformin, GW501516 and GW0742 increased memory performance and enhanced hippocampal neurogenesis as well.^370,384^ Thus, a phase IIa clinical study was carried out to test T3D-959, a newly synthetic PPARδ agonist, in subjects with mild to moderate Alzheimer’s disease, which suggests that PPARδ agonists are also moving towards clinical translation.^385^

**Fig. 7:**
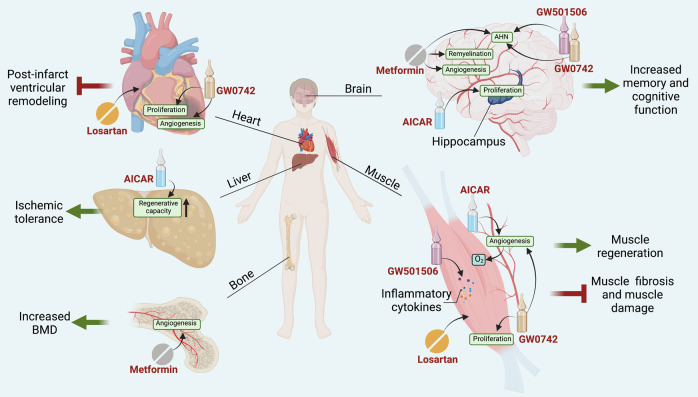
Emerging pharmaceuticals mimicking exercise-induced tissue regeneration. AMPK/SIRT1/PGC-1α/PPARδ pathway has been selected as an important intervention of exercise-induced physiological responses. AICAR, metformin, GW501516, and GW0742, used to mimic exercise-induced physiological responses, demonstrate the ability to promote damaged tissue repair. Additionally, losartan is used to promote muscle regeneration. Created with BioRender

Angiotensin II receptor blockade, an anti-hypertensive agent, is also an impressive replacement of exercise-induced regeneration. Losartan, a classical angiotensin-receptor blockade, was reported to limit post-infarct ventricular remodeling in rats, predominantly mimicking the protective effect of exercise on the heart.^386^ More recently, losartan reversed allodynia, reduced muscle fibrosis, and improved muscle regeneration in a murine model of orthopedic trauma combining tibia fracture and pin fixation with muscle damage, recapitulating the exercise-induced regeneration on post-injury recovery^387^ (Fig. 7).

### Natural molecular mediators

As mentioned above, the organism will release various natural molecular mediators involved in signaling pathways that promote regeneration during exercise. The regulatory mechanisms of these biomolecules are being understood by a wide range of researchers, which allows biosynthetic agents or genetic drugs to mimic the effects of exercise as well.

Irisin, a novel myokine, cleaved from membrane precursor fibronectin type III domain-containing 5 (FNDC5) in response to exercise, and acts as a linkage between muscles and other tissues.^388^ Besides inducing browning of white adipose tissue, irisin was discovered to promote proliferation and differentiation of osteoblasts through activating the p38 and ERK signaling.^389,390^ In the meantime, recombinant irisin positively regulated osteoblast differentiation under simulated microgravity via the overexpression of β-catenin, successfully providing a prevention strategy for bone loss and muscle atrophy induced by microgravity.^391^ Interestingly, irisin was also reported to bind to the proteins of the αV class of integrins, increasing production of SOST involved in bone resorption by increasing osteoclasts activity, which implied the two-sided effects of irisin on the therapeutic potential of skeletal remodeling.^392^ In terms of neuroprotection, circulating irisin crossed the blood-brain barrier and elevated BDNF level in the hippocampus, thereby promoting neurogenesis, enhancing synaptic plasticity, and modulating inflammation, which was demonstrated in models of neurodegenerative disease and cerebral ischemia.^393–395^ Furthermore, a recent study showed that recombinant adenovirus containing the irisin sequence improved burn-related neuropathy by ameliorating neuroinflammation-induced neuronal apoptosis, which demonstrated the protective effect of irisin on the PNS as well.^396^ Therefore, the therapeutic mechanism of irisin is not fully understood but has strong potential.

Adiponectin, one of the emerging adipokines, is also modulated by exercise, thus exerting a regenerative effect. Chronic exercise training imposed to rodents increased circulating adiponectin levels and AdipoR1 (adiponectin specific muscle receptor) expression.^397,398^ Thus, adiponectin was found to be responsible for the exercise-induced restoration of satellite cell mobilization, regenerative capacity in aged mice via the AMPK/SIRT1/PGC-1α axis.^399^ Moreover, adiponectin is capable of crossing the blood-brain barrier and affecting the CNS. Adiponectin mimicked exercise-induced effects in stress-elicited depression mice by retaining the normal proliferation of neural progenitors and dendritic morphology of neurons in the hippocampal dentate gyrus.^400^ In addition, AdipoRon (adiponectin receptor agonist), which mimicked the effects of running, rescued impaired cognitive function by improving hippocampal neurogenesis via adiponectin-Notch pathway.^401^ Similarly, AdipoRon treatment confirmed the exercise-induced hippocampal neuroplasticity in diabetic mice as well, which provides another promising candidate exercise mimetics.^402,403^

**Fig. 8:**
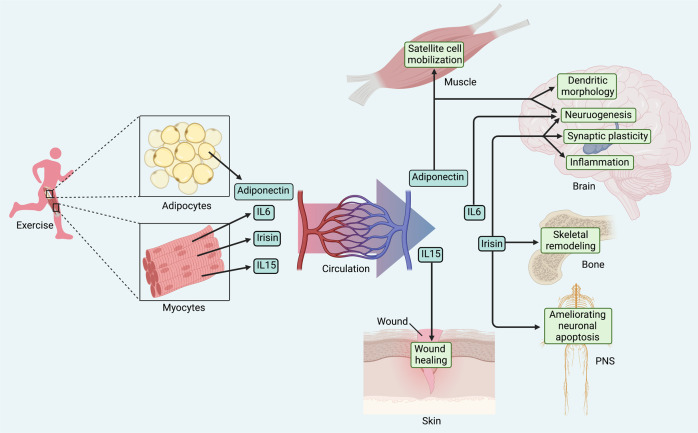
Natural molecular mediators. A set of exerkines are considered as candidate exercise mimetics, including irisin, adiponectin and interleukin, which can be used as gene therapy. These emerging target molecules provide new insights into the mechanisms of injured tissue regeneration and facilitate the efficiency of clinical translation. Created with BioRender

There are a set of immunomodulatory cytokines secreted into the circulation during exercise, such as interleukin-6 (IL-6) and IL-15, which are also integrated into the list of candidate exercise mimetics. IL-6 has been found to be synthesized and secreted into circulating by skeletal muscle during exercise, activating PI3K/Akt signaling, MAPK signaling and AMPK signaling in targeting cells.^404–406^ IL-6 was shown to promote proliferation of post-natal murine neural stem cell numbers.^407^ Thus, administration of recombinant IL-6 in a low-dose pulsatile strategy might directly modulate Schwann and nerve cells as a regenerative response to exercise in diabetic peripheral neuropathy.^408^ Notably, tocilizumab, one of the IL-6 receptor antibodies, has been used to treat some forms of arthritis.^409^ The side effect, such as blocking of exercise-mediated loss of visceral adipose tissue mass, has been confirmed recently.^410,411^ Whether the application of IL-6 receptor antibodies will block exercise-induced regenerative capacity is also a topic worthy of further investigation. While IL-15 is also a myokine of the IL-2 family responding to exercise.^412^ IL-15 has been reported to have local effects on skeletal muscles, such as promoting myoblast differentiation.^413,414^ Furthermore, exercise-mediated improvements in the healing of aged skin depend upon circulating IL-15. Exercise-mimicking recombinant IL-15 directly enhanced the growth of the aged mouse fibroblasts and keratinocytes, promoting impaired wound healing via activation of signal transducer and activator of transcription 3 signaling pathway, even though it was barely scarless regeneration^415^ (Fig. 8).

## Discussion and perspective

### Application of exercise intervention under aging condition

With the progress of society, the average human life expectancy has increased significantly. In parallel with longer lifespan, aging-specific health problem have emerged, which makes a growing global burden. Although it is brought to light that aging can not to be abolished, it is still expected to be able to attenuate the process and greatly ameliorate its effects, which implies the ultimate goal is not only longer lifespan, but also a better quality of life.^416^ Indeed, besides trauma and inflammation, aging is a vital process in the lifespan, whose essence is replicative cellular senescence.^417^ Senescent cells accumulate at sites of age-related pathologies and have an impact on the normal physiology of the tissues, causing a progressive functional deterioration.^418^ Exercise training is considered as a promising regenerative intervention for aged tissues, contributing to prevention and management of the challenging chronic diseases faced by elderly population.

Dementia is characterized by impairment of cognitive abilities and memory. Alzheimer’s disease (AD), a main and common type of dementia, is probably identified as age-related impairment of AHN.^143^ It has been reported that exercise not only reduces the risk of developing AD in aged population, but also improves the cognitive function of individuals with mild cognitive impairment and AD.^419^ Increasing evidence suggests that a variety of bioactive substances induced by exercise exert neuroprotective effects in mouse models via restoration of AHN and regulation of synaptic plasticity as well.^420,421^ In addition, numerous studies have shown that exercise can improve the poor prognosis of other age-related neurodegeneration via promoting neuronal survival and plasticity, or neurogenesis.^422^ Interestingly, endurance exercise has been reported to enhance the secretion of an enzyme, called glycosylphosphatidylinositol-specific phospholipase D1, derived from liver, ameliorating impaired neurogenesis and cognition in the aged hippocampus of mice.^423^ It is a new approach to explore the influence of other organs on the aged brain, providing new targets for the treatment of neurodegenerative diseases.

The level of bone loss increases with age, leading to osteoporosis in the elderly, particularly postmenopausal woman, which increases risk of fractures. Exercise training is considered as an effective method to stimulate bone osteogenesis in osteoporotic patients. As is mentioned, bone is a force-receptive organ, which needs to achieve sufficient mechanical intensity to effectively trigger the response of osteogenesis. As a result, not all types of exercise have the same positive effect on BMD. High intensity aerobic or resistance exercises are confirmed to be more effective in promoting the increase of BMD.^424,425^ In addition, several meta-analyses have shown that exercise appears extremely site-specific, increasing BMD only in the stimulated body regions.^426,427^ Thus, more composite exercises of diverse patterns and intensities need to be explored in order to more effective bone regeneration in whole body of aging adults.

Sarcopenia, the loss of skeletal muscle mass and strength, is an inevitable event during the aging process, which reduces physical capacity and enhances the problems associated with disabilities.^428^ Capelli et al.^429^ indicated that decay of maximal aerobic power and anaerobic capacity occurred with aging in cycling athletes, confirming age-related loss of muscle mass. Accumulating evidence supports that exercise training represents an effective intervention strategy to reduce or even reverse age-related loss of muscle mass as well.^430^ A meta-analysis, including 1,328 adults, demonstrated that resistance exercise training was effective in eliciting gains in lean body mass among the older people, particularly if they performed higher volume programs.^431^ Thus, as an effect of the independent exercise regimes on muscle mass, resistance exercise programs seem to be mostly effective in increasing muscle strength in sarcopenic frail elderly people.^432,433^ In regard of the cellular level, both resistance and endurance exercise training have shown to increase the number of satellite cells for regeneration in old animals and humans.^434–437^ The key role for muscle regeneration may be the intensity and frequency of exercise stimulation, however, the specific mechanisms responsible for re-trigger of growth capacity by exercise are not of comprehensive recognition yet.

### Limitation and prospects of exercise intervention

Although remarkable progress has been made in the treatment of exercise interventions over the past few decades (Table 1), the side effects of over exercise are also being recognized. It is common to visualize that excessive-exercise or inappropriate exercise leads to sport-related injuries, ranging from the ankle and the knee, to the face and even the brain, which has ruined the careers of most athletes.^438–441^ Notably, chronic excessive exercise might adversely impact cardiovascular health. The increased incidence of atrial fibrillation seen in endurance athletes is one of the best documented cardiac maladaptations, which is related to exercise-induced changes in autonomic tone alongside the development of an arrhythmogenic atrial substrate.^442^ Myocardial fibrosis and coronary artery calcification have also been detected in ultra-endurance races.^443,444^ In addition, the marked suppression of growth factors and hormones, including testosterone, IGF-1, and leptin, after ultra-endurance exercise has also reported, which is strongly associated with the magnitude of the energy deficit.^445^ It has been realized that excessive exercise leads to immune imbalance and decrease in reactive oxygen species scavenging capacity, which has deleterious effects on health as well.^446^ Fortunately, with a better understanding of the adaptive responses of the organism to exercise gained, it has been found that we all carry our own “endogenous medicine box”. We have the opportunity to take the most applicable pills from the box to target a variety of different diseases. Thus, how to explore and make good use of the body’s own endogenous health resources, especially how to develop personalized rehabilitation exercise prescriptions for different diseases and different patients, has attracted more and more researchers to explore this field.

**Table 1:** Clinical trials related to exercise intervention in diverse diseases

Organ	Disease	Participants	Intervention	Course	Effect	Registration number	Reference
Muscle	Sarcopenia	Adult women aged over 65 years with sarcopenia	Body weight-based and elastic band resistance exercise	60 min/session, 3 times/week, for 16 weeks	Grip strength↑, gait speed↑, isometric muscle strength↑	The Institutional Review Board (KHSIRB-18-021)	Seo et al.^447^
		Older men with osteoporosis and sarcopenia	Low-volume/high-intensity-dynamic resistance exercise	Twice/week, for 12 months	Integral lumbar spine BMD↑, SMI↑, maximum hip/leg extensor strength↑	NCT03453463	Kemmler et al.^448^
		Adults with liver cirrhosis	Progressive resistance exercise training	60 min/session, 3 times/week, for 12 weeks	Peak torque in isokinetic knee extension↑, CSA of the quadriceps muscle↑	NCT02343653	Aamann et al.^449^
		Older men with osteosarcope	High-intensity resistance exercise training	Twice/week, for 28 weeks	Sarcopenia *Z*-score↑, SMI↑, handgrip strength↑	NCT03453463	Lichtenberg et al.^450^
Bone	Osteoporosis	Postmenopausal women with osteoporosis	Exercise program consisting of resistance exercise, balance exercise and aerobic exercise	Aerobic exercise: rapid walking, 60 min/day, 5 days/week, for 12 weeks resistance exercise and balance exercise: 70 min/day, 3 times/week, for 12 weeks	Muscle strength↑, walking performance↑, static balance↑	NCT03816449	Filipović et al.^451^
		Postmenopausal women with osteoporosis	High-intensity resistance and impact training, home-based exercise	30 min/day, twice/week, for 8 months	Inclinometer-determined standing tall thoracic kyphosis↑, indices of bone strength↑, functional performance↑	ACTRN12616000475448	Watson et al.^424,452^
		Adult men aged over 45 years with osteoporosis	High-intensity resistance and impact training, supervised machine-based isometric axial compression	30 min/day, twice/week, for 8 months	Femoral neck BMD↑, area and bone strength index↑, and trabecular BMC↑and bone strength index↑	ANZCTR12616000344493	Harding et al.^453^
		Postmenopausal women with osteoporosis	Exercise program contains low intensity strength and balance exercise	60 min/day, 3 times/week, for 6 months	Static balance↑, dynamic balance↑, strength of the upper and lower limbs↑	Landako Health Center in the Basque Country (Northern Spain)	Otero et al.^454^
Heart	Heart failure	Frail adults with HFrEF	Supervised aerobic exercise training: cycle or walk-based exercise	>30 min/day, 3 times/week, for 3 months	Risk of all-cause hospitalization↓	NCT00047437	Pandey et al.^455^
		Middle-aged adults with LVH	Combination of yoga, balance, and strength training	3 times/week, for 12 months	VO_2_ max↑, LV myocardial stiffness↓	NCT03476785	Hieda et al.^456^
Brain	SIVCI	Adults with mild SIVCI	Progressive aerobic training	3 times/week, for 6 months	General cognitive function (ADAS-Cog performance)↑, 6-minute walk distance↑	NCT01027858	Liu-Ambrose et al.^457^
	Stroke	Stroke adults with vascular cognitive impairment	Combination of endurance, strength, and balance exercise	50 min/day, 3 times/week, for 3 months	Cognitive performances↑	ISRCTN 16009172	Wang et al.^458^
		Adults with subacute stroke	Aerobic, body weight supported, treadmill based physical fitness training	25 min/day, 5 times/week, for 4 weeks	Maximal walking Speed→, Barthel index score→, adverse events↑	NCT01953549.	Nave et al.^459^
	Alzheimer’s disease	Adults with mild–moderate Alzheimer’s disease	Moderate-to-high-intensity aerobic exercise	60 min/day, 3 times/week, for 16 weeks	Basic mobility↑, usual gait speed↑, fast gait speed↑	NCT01681602	Sobol et al.^460^
		Adults with subjective memory impairment	Moderate-plus activities included moderate, hard, and very hard intensity activities (e.g., brisk walking, ballroom dancing, gym circuit, or swimming)	50 min/day, 3 times/week, for 18 weeks	General cognitive function (ADAS-Cog performance)↑	ACTRN12605000136606	Lautenschlager et al.^461^
	Dementia	Adults with mild-to-moderate dementia	Moderate to high intensity aerobic and strength exercise training	60–90 min/day, 3 times/week, for 16 weeks	Physical fitness (6 min walk test)↑, general cognitive function (ADAS-Cog performance)→	ISRCTN10416500	Lamb et al.^462^

*BMD* bone mineral density, *SMI* skeletal muscle mass index, *CSA* cross-sectional area, *BMC* bone mineral content, *HFrEF* heart failure with reduced ejection fraction, *LVH* left ventricular hypertrophy, *VO*_*2*_ maximal oxygen uptake, *SIVCI* subcortical ischemic vascular cognitive impairment, *ADAS-Cog* Alzheimer’s Disease Assessment Scale–Cognitive subscale

As a matter of fact, the knowledge of the adaptive responses to exercise is still only the tip of the iceberg. There are still three major points in the current researches on exercise for regeneration requiring further exploration. Firstly, the organism responds differently to diverse exercise patterns and intensities with great individual variability, resulting in instability and poor reproducibility of the exercise test. Therefore, the design of a rational exercise intervention is helpful to investigate the mechanisms of exercise regeneration, which is more effectively applied in clinical treatment further. Secondly, although the role of exercise for tissue regeneration undoubtedly brings new ideas and strategies, exercise mimetics need in-depth exploration. Notably, exercise mimetics may have utility across a wide range of human disorders, which is a gift for patients who are subjectively or objectively unable to achieve exercise benefits. However, as individual variability in exercise, exercise mimetics are not going to work as a universal panacea for divergent disorders but are more likely to be most effective for specific disorders, or even subtypes of such disorders. Thus, exploration of multi-target exercise mimetics is a key step in broadening the range of applications and improving the value of clinical translation. Thirdly, regeneration is indeed an anti-aging remedy, but it also often goes hand in hand with tumors. Whether exercise mimetics have a carcinogenic risk while promoting cell proliferation deserves more research to prove, which means a higher demand on the administration and dose of the drugs.

Beyond any doubt, exercise-induced regenerative medicine is an emerging and promising discipline. Currently, a variety of signaling pathways and related novel biomolecules have been identified in exercise adaptive regeneration, exhibiting more potential perspectives for disease prevention and treatment.
